# Induction of autoantibodies against lung matrix proteins and smoke-induced inflammation in mice

**DOI:** 10.1186/1471-2466-10-64

**Published:** 2010-12-13

**Authors:** Corry-Anke Brandsma, Wim Timens, Marie Geerlings, Henrike Jekel, Dirkje S Postma, Machteld N Hylkema, Huib AM Kerstjens

**Affiliations:** 1 Department of Pathology and Medical Biology, University Medical Center Groningen, University of Groningen, Groningen, the Netherlands; 2 Department of Pulmonary Diseases, University Medical Center Groningen, University of Groningen, Groningen, the Netherlands

## Abstract

**Background:**

Smoking is the major etiologic factor in COPD, yet the exact underlying pathogenetic mechanisms have not been elucidated. Since a few years, there is mounting evidence that a specific immune response, partly present as an autoimmune response, contributes to the pathogenesis of COPD. Increased levels of anti-Hep-2 epithelial cell and anti-elastin autoantibodies as well as antibodies against airway epithelial and endothelial cells have been observed in COPD patients. Whether the presence of these autoantibodies contributes to the pathogenesis of COPD is unclear.

**Methods:**

To test whether induction of autoantibodies against lung matrix proteins can augment the smoke-induced inflammatory response, we immunized mice with a mixture of the lung extracellular matrix (ECM) proteins elastin, collagen, and decorin and exposed them to cigarette smoke for 3 or 6 months. To evaluate whether the immunization was successful, the presence of specific antibodies was assessed in serum, and presence of specific antibody producing cells in spleen and lung homogenates. In addition, the presence of inflammatory cells and cytokines was assessed in lung tissue and emphysema development was evaluated by measuring the mean linear intercept.

**Results:**

We demonstrated that both ECM immunization and smoke exposure induced a humoral immune response against ECM proteins and that ECM immunization itself resulted in increased macrophage numbers in the lung. The specific immune response against ECM proteins did not augment the smoke-induced inflammatory response in our model.

**Conclusions:**

By demonstrating that smoke exposure itself can result in a specific immune response and that presence of this specific immune response is accompanied by an influx of macrophages, we provide support for the involvement of a specific immune response in the smoke-induced inflammatory response as can be seen in patients with COPD.

## Background

Chronic Obstructive Pulmonary Disease (COPD) is a leading cause of death worldwide with an increasing morbidity and mortality [1]. Smoking is the most important risk factor for the development of COPD and smoking cessation is currently the most effective treatment to diminish the accelerated lung function decline associated with COPD. The exact pathogenetic mechanisms underlying the smoke-induced chronic inflammatory response in the lungs of COPD patients are still largely unclear and this hampers the search for new and more effective treatment strategies.

Since a few years, there is mounting evidence that a specific immune response, partly present as an autoimmune response, contributes to the pathogenesis of COPD. Oligoclonal CD4 T cells have been demonstrated in lung tissue of severe COPD patients [2] as well as an antigen specific Th1 response against lung elastin [3], indicating an antigen specific T cell response in COPD. Additionally, B cells organized into lymphoid follicles have been demonstrated in lung tissue of COPD patients [4] and the number of follicle containing airways increases with disease severity [5]. Vh gene analysis of these B cells showed oligoclonality and somatic hypermutations [4] supporting the presence of an antigen specific B cell response in COPD.

It is not clear against which antigen (s) this specific immune response in COPD is directed. We considered three potential sources of antigens; 1) microbial antigens, 2) cigarette smoke components or derivatives, or 3) auto antigens derived from degradation products of the extracellular matrix [6]. We recently showed increased percentages of class switched memory B cells in peripheral blood of current smokers with or without COPD compared to never and ex-smokers [7]. These findings suggest the presence of an ongoing smoke-induced specific immune response. With respect to auto antigens, a high prevalence of autoantibodies against Hep-2 epithelial cell has been reported in COPD [8,9] as well as a high prevalence of autoantibodies against airway epithelial cells [8], endothelial cells [10], lung elastin [3], several immunogenic peptides [11] and cytokeratin 18 [12].

We propose that neo-antigens may arise during the chronic inflammatory response in COPD due to lung tissue destruction and/or continued smoke exposure. These neo-antigens are recognized and evoke an antigen specific immune response, characterized by antigen specific T-and B cells in the lung, organized into lymphoid follicles and the presence of autoantibodies. We question however, whether the presence of this specific immune response subsequently augments the chronic inflammatory response in COPD, thereby causing more severe disease and autoimmunity, or whether it is just an epiphenomenon of the ongoing inflammation and destruction. To answer this question, autoimmune animal models for COPD have to be developed. Taraseviciene-Stewart *et al *demonstrated that a specific anti-endothelial immune response can induce emphysema in an experimental animal model [13]. However, they did not investigate whether this anti-endothelial immune response can enhance the smoke-induced inflammatory response and emphysema.

To test whether the induction of antibodies against lung matrix proteins can augment the smoke-induced immune response, we immunized mice with a mixture of mouse decorin, lung elastin, and lung collagen and exposed them to cigarette smoke for 3 or 6 months. The levels of specific antibodies and antibody producing cells were investigated as well as the levels of inflammatory cells and cytokines and the development of emphysema.

## Methods

### Study design

Female C57BL/6 mice (Harlan, Zeist, the Netherlands) were divided into seven groups containing 8-12 animals per group (Table 1). The mice were immunized with extracellular matrix proteins (ECM) and adjuvant or with adjuvant alone and subsequently exposed to cigarette smoke or sham exposure for 3 or 6 months. An extra control group was added for the 3 months of exposure to study the effect of ECM immunization separately. This group received ECM immunization alone and was not exposed to cigarette smoke.

**Table 1:** Groups with different treatments and exposures

	Group	Immunization	Exposure
**3 months of exposure**	Control Smoke	Adjuvant	Smoke
	
	ECM Smoke	ECM + Adjuvant	Smoke
	
	Control Sham smoke	Adjuvant	Sham smoke
	
	ECM Sham smoke	ECM + Adjuvant	Sham smoke

**6 months of exposure**	Control Smoke	Adjuvant	Smoke
	
	ECM Smoke	ECM + Adjuvant	Smoke
	
	Control Sham smoke	Adjuvant	Sham smoke

Before, during and at the end of the experiment blood was obtained via orbital punctures to measure serum levels of ECM specific antibodies. The smoke exposure protocol started one week after the last immunization booster. Mice were sacrificed after 3 or 6 months of smoke or sham smoke exposure. The trachea was cannulated, the right lung was ligated and lung lobes were snap-frozen and stored at -80°C for immunohistochemical or cytokine analyses. The left lung was inflated and fixed for 24 h with formalin with a constant pressure of 25 cm H_2_O for emphysema measurements or freshly used for leukocyte isolation to perform ELISPOT analysis. The spleen was removed to freshly isolate leukocytes for ELISPOT analysis. Experiments were approved by the Institutional Animal Care and Use Committee of the University of Groningen (IACUC-RuG).

### Immunization procedure

Mice were immunized intraperitoneally with 100 μl of Sigma Adjuvant (Sigma-Aldrich, St. Louis, USA) alone or in combination with 10 μg mouse lung collagen (Elastin Products, Owensville, USA), 10 μg recombinant mouse decorin (R&D Systems, Minneapolis, USA) and 10 μg mouse lung elastin peptides (Elastin Products). Three and 6 weeks after the first immunization a booster immunization was given with the same mixtures.

### Smoke exposure

Mice were exposed nose only to 24 puffs of cigarette smoke from two 2R1 reference cigarettes (University of Kentucky) two times per day, for 5 days a week for 3 or 6 months, as described previously [4,14]. Sham exposed mice were exposed to air under the same conditions.

### ELISA for ECM specific antibodies

To measure serum levels of ECM specific antibodies, 96-well plates (Nunc maxisorp, Thermo Fisher Scientific, Denmark) were coated overnight at 4°C with collagen (10 μg/ml), decorin (10 μg/ml) or elastin (25 μg/ml) (similar proteins as used for the immunization). Non specific binding was blocked with 0.2% skim milk in PBS for 1.5 h at 37°C. Plates were then incubated with 50 μl of diluted serum for 2 hours at 37°C, followed by rat-anti-mouse IgG1-biotin (BD Pharmingen, San Diego, USA) or goat-anti-mouse IgM-biotin (Southern Biotechnology, Birmingham, USA) for 2 hours at 37°C and AB complex (Dako, Glostrup, Denmark) for 30 min at room temperature. The presence of ECM specific antibodies was visualized using 3,3,5,5, tetramethylbenzidine (TMB) substrate (Sigma) and analyzed with the Varioskan microplate reader (Thermo Fisher Scientific, Waltham, USA) using SkanIt software. One sample with a high optical density value (OD) was titrated in serial dilutions to obtain a standard curve. This standard curve was used to convert the obtained OD values into relative units.

### Cell isolation and ELISPOT analysis

Single cell leukocytes suspensions were freshly isolated from spleen and lung tissue. Spleens were grinded between two microscopy slides and cell homogenates were filtered to obtain single cell suspensions. Single cell lung suspensions were obtained as described previously [15]. Briefly, lungs were minced with a razor blade and incubated in a shaking water bath for 90 min at 37°C in digestion buffer containing RPMI 1640 medium supplemented with 10% fetal calf serum (FCS) (both Lonza, Verviers, Belgium), DNAse I (100 U/ml; Boehringer, Mannheim, Germany), and collagenase I (250 U/ml; Sigma-Aldrich). The digested lung tissue was passed through a 70 μm cell strainer (BD) and subsequently centrifuged on a Percoll gradient (20-55%; GE Healthcare, Uppsala, Sweden). Cells were counted using a Sysmex PocH-100i cell counter (Sysmex, Roche, Germany).

For the ELISPOT analysis, 96-Wells plates (Nunc maxisorp) were coated overnight at 4°C with collagen, decorin or elastin (similar concentrations as used for ELISA). The plates were washed with PBS/0.05%Tween and blocked with 4% PBS/BSA. Isolated spleen and lung cells (concentrations ranging from 0.25-1.10^6 ^cells) were added to each well and incubated overnight at 37°C with 5% CO_2_. The cells were removed by vigorous washing with PBS followed by PBS/0.05%Tween. The number of ECM specific antibody producing cells that were present in spleen en lung tissue was identified by adding rat-anti-mouse IgG1-biotin (BD Pharmingen) or goat-anti-mouse IgM-biotin (Southern Biotechnology) followed by Streptavidin-Alkaline Phosphatase (Dako) and visualized as blue spots by using BCIP in agarose (both Sigma). The number of spots was counted per well and expressed as number of spots per million cells.

### Histology

Total numbers of macrophages and neutrophils were identified in frozen lung tissue with specific antibodies against CD68 (AbD Serotec, Düsseldorf, Germany) and Gr-1 (BD Pharmingen). B cell and plasma cell numbers were identified in formalin fixed and paraffin embedded lung tissue with antibodies against B220 (BD Pharmingen) and CD138 (BD Pharmingen) respectively. Total cell numbers were counted at 25x magnification and expressed as the number of positive cells per total surface area lung tissue, which was assessed by morphometric analysis using Leica Qwin image analysis software (Leica Microsystems BV, Rijswijk, the Netherlands).

The expression of collagen and decorin was identified in formalin fixed and paraffin embedded lung tissue with a Picro-Sirius red staining to visualize collagen and a specific antibody against decorin (kind gift from Larry Fisher, Bethesda, USA). The expression of elastin was identified in frozen lung tissue with a specific antibody against elastin (Cedarlane, Ontario, Canada). Collagen bundles were visualized using polarized light. The deposition of these extracellular matrix proteins in lung tissue was scored semi-quantitatively.

### Morphometrical evaluation of emphysema

Alveolar airspace enlargement was assessed on the formalin fixed and inflated lungs by mean linear intercept (Lmi) by two independent individuals in a blinded manner, as described previously [4,16].

### Cytokines

Frozen lung tissue was homogenized in 50 mM Tris-HCl buffer containing 150 mM NaCl and 0.002% Tween-20 (pH 7.5) and centrifuged at 12000g for 10 min to remove any insoluble material. Concentrations of IFN-γ, IP-10, FGF basic, MIP-1α, MCP-1, VEGF, IL-5, IL-2, GM-CSF, TNF-α, IL-10, IL-12, IL-13, IL-17, IL-1α, IL-1β, IL-4, IL-6, KC and MIG in supernatants were measured with a Biosource Mouse Cytokine 20 plex kit (Invitrogen, Breda, the Netherlands) using a multiplex ELISA system (Lincoplex Systems, St Charles, MO, USA).

### Statistics

Mann Whitney U tests were used to compare the difference between the groups. A value of p < 0.05 was considered significant.

## Results

### ECM immunization and smoke exposure increase serum levels of specific antibodies

**Figure 1:**
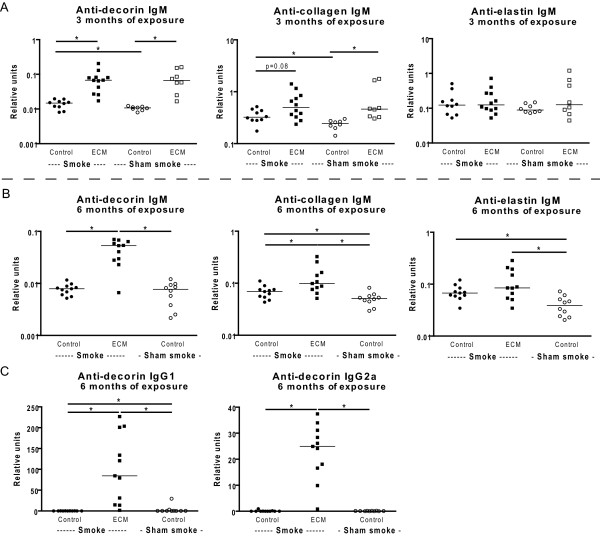
**Serum levels of ECM specific antibodies**. Serum levels of IgM (A,B), and IgG1 and IgG2a (C) antibodies against decorin, collagen and elastin are depicted. In A) the levels of the ECM specific antibodies are shown for the 3 months exposed mice. In B) and C) the levels of ECM specific antibodies are shown for the 6 months exposed mice. Below the figures the different exposures are indicated; Control = control immunization with adjuvant, ECM = immunization with ECM proteins, Smoke = smoke exposure, Sham smoke = control exposure to air. Smoke exposed mice are indicated with black symbols and sham smoke exposed mice with open symbols. * indicates p < 0.05.

After 3 months of exposure, increased levels of anti-decorin and anti-collagen IgM antibodies were present in ECM immunized mice. This ECM effect was present in both smoke- and sham smoke exposed mice (Figure 1A). There was no effect of ECM immunization on the levels of anti-elastin IgM antibodies in the 3 months exposed mice.

After 6 months of exposure, increased levels of anti-decorin and anti-collagen IgM antibodies were present in ECM immunized smoking mice when compared to control immunized smoking mice or control immunized sham smoke exposed mice (Figure 1B). For elastin, there was only an increase in IgM antibody levels in ECM immunized smoking mice when compared to control immunized sham smoke exposed mice. For decorin, there were also increased levels of IgG1 and IgG2a antibodies present in the ECM immunized smoke exposed mice when compared to control immunized smoking mice or control immunized sham smoke exposed mice (Figure 1C).

Additionally, smoke exposure itself increased the levels of IgM antibodies against collagen and decorin after 3 months of exposure and against collagen and elastin after 6 months of exposure.

### ECM immunization and smoke exposure increase the numbers of specific antibody producing cells in spleen and lung

**Figure 2:**
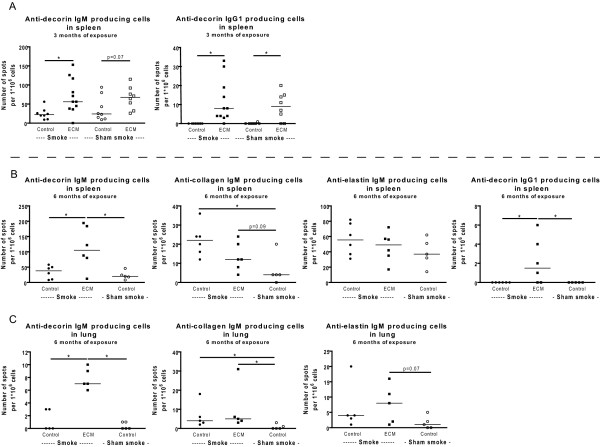
**ECM specific antibody producing cells**. The numbers of IgM (A,B,C) and IgG1 (B) antibody producing cells against decorin, collagen and elastin in spleen (A,B) and lung (C) are depicted. In A) the numbers of anti-decorin IgM and IgG1 antibody producing cells in spleen are shown for the 3 months exposed mice. In B) the numbers of anti-decorin, -collagen -and elastin IgM antibody producing cells and anti-decorin IgG1 antibody producing cells in spleen are shown for the 6 months exposed mice. In C) the numbers of anti-decorin, -collagen -and elastin IgM antibody producing cells in lung are shown for the 6 months exposed mice. * indicates p < 0.05.

After 3 months of exposure, increased numbers of anti-decorin IgM and IgG1 producing cells were present in the spleen of ECM immunized mice. This ECM effect was present in both smoke exposed and sham smoke exposed mice (trend for IgM) (Figure 2A). There was no effect of ECM immunization on the numbers of anti-elastin IgM producing cells and anti-collagen IgM producing cells could not be quantified in the 3 months exposed mice.

After 6 months of exposure, increased numbers of anti-decorin IgM and IgG1 producing cells were present in both spleen and lung (only IgM) of ECM immunized smoking mice when compared to control immunized smoking mice or control immunized sham smoke exposed mice (Figure 2B, C). For collagen there was an increase in IgM antibody producing cells in the lung and spleen (trend) in the ECM immunized smoking mice when compared to control immunized sham smoke exposed mice and in the lung there was a similar trend for elastin. Additionally, 6 months of smoke exposure increased the numbers of anti-collagen IgM producing cells in both spleen and lung.

### Smoking increases the numbers of macrophages, B cells and plasma cells in lung tissue; no additional effect of ECM immunization

**Figure 3:**
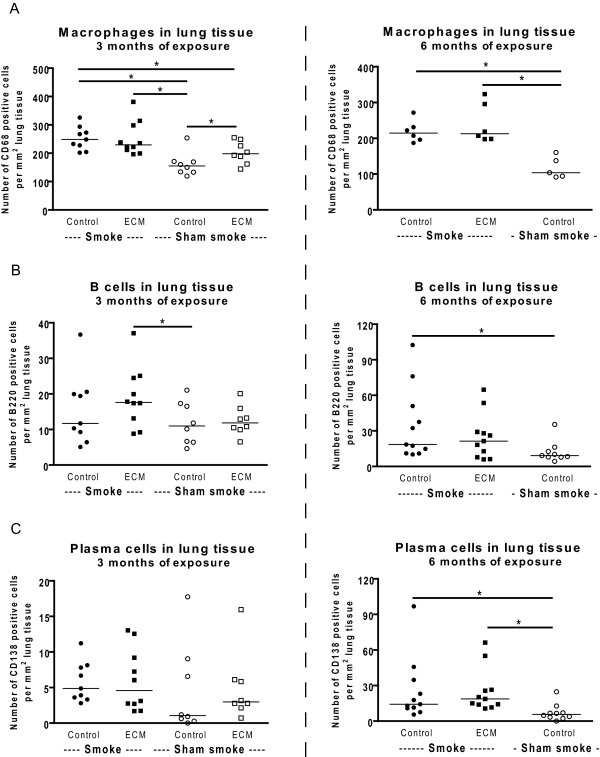
**Inflammatory cells in lung tissue**. The numbers of macrophages (A), B cells (B) and plasma cells (C) in lung tissue are shown for the 3 (left) and 6 months (right) exposed mice. * indicates p < 0.05.

Both 3 and 6 months of smoke exposure caused an increase in the numbers of macrophages in lung tissue (Figure 3A, control immunized smoking mice and ECM immunized smoking mice compared to control immunized sham smoke exposed mice). In addition, the results of the 3 months exposed mice show that ECM immunization alone also increased the numbers of macrophages in lung tissue (control immunized and sham smoke exposed compared to ECM immunized and sham smoke exposed). There was no additional effect of ECM immunization on the numbers of macrophages in lung tissue compared to the effect of smoking alone (control immunized smoking mice compared to ECM immunized smoking mice).

Six months of smoke exposure caused an increase in the numbers of B cells and plasma cells in lung tissue (Figure 3B and 3C, control immunized smoking mice compared to control immunized and sham smoke exposed mice). This effect was not present after 3 months of smoke exposure. There was no (additional) effect of ECM immunization on the presence of B cells and plasma cells in lung tissue (control immunized smoking mice compared to ECM immunized smoking mice). There were no effects of smoking or ECM immunization on the numbers of neutrophils in lung tissue (data not shown).

### Smoking increases collagen deposition; no additional effect of ECM immunization

**Figure 4:**
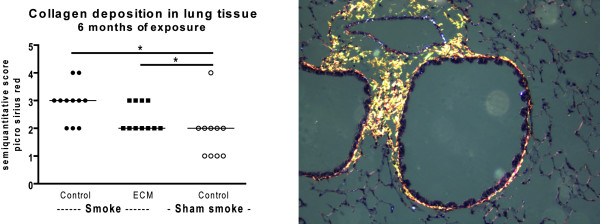
**Collagen deposition in lung tissue**. The semi quantitative score for the collagen deposition in lung tissue is shown for the 6 months exposed mice (left). * indicates p < 0.05. On the right an example of collagen deposition in lung tissue is shown.

After 6 months of smoke exposure there was an increase in collagen deposition around vessels and airways in the lungs of both control immunized and ECM immunized smoking mice (Figure 4). There were no effects of smoking or ECM immunization on decorin and elastin deposition in the lung (data not shown).

### No effect of smoking and ECM immunization on emphysema development

**Figure 5:**
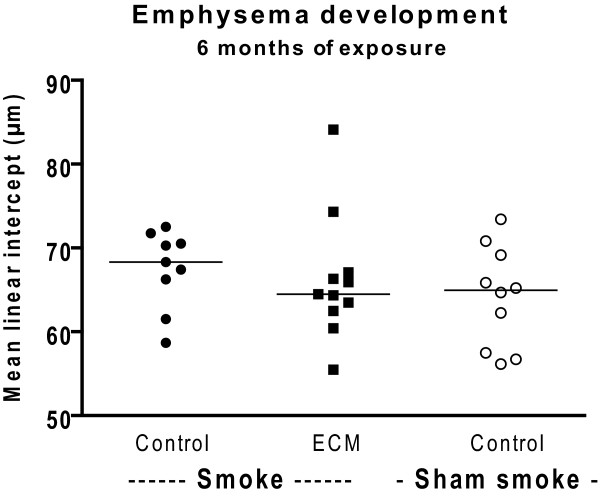
**Emphysema development**. The mean linear intercept score, a measure for emphysema, is shown for the 6 months exposed mice.

After 6 months of smoke exposure there was no significant emphysema development, nor was there an (additional) effect of ECM immunization (Figure 5).

### Smoking increases the levels of IL-1α and IL-12 in lung tissue and ECM immunization increases the levels of FGF basic and VEGF

**Figure 6:**
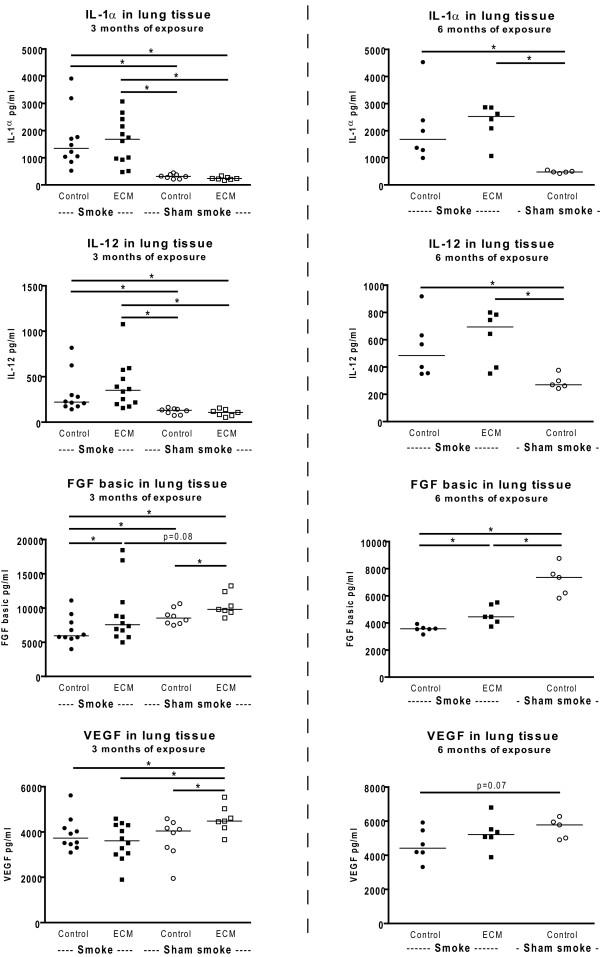
**Cytokine levels in lung tissue**. The levels of IL-1α, IL-12, FGF basic and VEGF in lung tissue are shown for the 3 (left) and 6 months (right) exposed mice. * indicates p < 0.05.

Both 3 and 6 months of smoke exposure increased the levels of IL-1α and IL-12 in lung tissue (Figure 6 control immunized smoking mice compared to control immunized and sham smoke exposed mice). In the 3 months exposed mice this smoke effect is also present in the ECM immunized mice (ECM immunized smoking mice compared to ECM immunized sham smoke exposed mice). There was no (additional) effect of ECM immunization on these cytokines.

In the 3 months exposed mice, ECM immunization increased the levels of FGF basic and VEGF in the sham exposed mice. For FGF basic this effect was also present in the 3 and 6 months smoke exposed mice. Additionally, 3 and 6 months of smoke exposure decreased the levels of FGF basic and there was a similar trend for VEGF. There were no effects of smoking or ECM immunization on IL-2, IL-10 and MIG (data not shown). The levels of GMCSF, IL-1β, IL-4, IL-5, IL-6, IL-13, IL-17, IP-10, KC, MCP-1, MIP-1α, and TNF-α in lung tissue were at or below the detection limit of the assay.

## Discussion

Our study shows that immunization with mouse lung decorin, elastin and collagen effectively increases serum levels of specific antibodies and antibody producing cells against these proteins in the spleen and lung. However, these specific antibodies and specific antibody producing cells did not augment the smoke-induced inflammatory response in our mouse model. Interestingly, smoke exposure also increased levels of specific antibodies in serum and anti-collagen antibody producing cells in spleen and lung, and ECM immunization increased numbers of macrophages in the lung.

The effect of immunization with lung matrix proteins on subsequent antibody production was most prominent for decorin. Immunization resulted in high IgM, IgG1 and IgG2a antibody levels in serum as well as IgM and IgG1 antibody producing cells in the spleen and IgM antibody producing cells in the lung. The presence of anti-decorin IgM antibody producing cells in the lung is of particular interest, since it reflects both systemic and local pulmonary effects of immunization. The latter may be crucial in contributing to the chronic inflammatory response in the lung and the diminished presence of decorin around the airways, as we previously demonstrated in patients with severe COPD [17]. The presence of a specific IgG response against decorin is a result of class switch recombination and suggests that the booster immunizations with decorin evoked a strong secondary immune response. The immune responses for collagen and elastin were less strong and we did not find evidence for class switch recombination.

In addition, we observed an increase in macrophage numbers in the ECM immunized mice that were not exposed to cigarette smoke. This is compatible with previous data showing increases in macrophage and neutrophil numbers and MMP9 and MMP12 levels in mice immunized with elastin peptides [18]. These findings indicate that the induction of a specific immune response against ECM proteins itself can evoke a "COPD like" inflammatory response in the lung.

Interestingly, smoke exposure itself also induced a specific IgM immune response against collagen and elastin, which is in line with our previous findings showing higher levels of class switched memory B cells in current smokers than in ex- and never smokers [7]. Both reflect a smoke induced specific immune response.

Against our expectations, the increased level of anti-ECM antibodies and antibody producing cells in the spleen and lung of ECM immunized mice was not associated with enhancement of the smoke-induced inflammatory response in this model. There can be several explanations for these findings. First, 6 months of smoke exposure did not evoke a very strong inflammatory response, i.e. no increase in neutrophils and no significant emphysema development. Therefore, one may argue whether the effects of smoking were strong enough to investigate if anti-ECM antibodies can augment the smoke-induced inflammatory response. We have used the same mouse strain, gender, housing conditions, and smoking model as before [4]. The only difference is that there were some years in between the two studies and thus a different batch of C57BL/6 mice was used and the cigarettes were older. Apart from that we do not have a clear explanation for the current lack of effect on inflammation and emphysema.

Secondly, we also observed a specific immune response against collagen and elastin in smoking mice, resulting in similar anti-collagen and anti-elastin immune responses as in the ECM immunized smoking mice. Thus, if the specific immune responses against collagen and elastin would have had an augmenting effect on the smoke-induced immune response, it might have been very difficult to detect. On the other hand, the immune response against decorin was very strong in the ECM immunized smoking mice and differed significantly from the smoking mice. Therefore, the lack of an effect of ECM immunization may indicate that a specific immune response against decorin does not augment the smoke-induced inflammatory response in this model. However, compared to the human situation, where differences in decorin expression and modulation of decorin production are particularly present in patients with severe COPD [17,19], one could still argue that the effect of the specific anti-decorin response might be present after longer smoke exposure or in a model with stronger effects of smoking.

Finally, the choice of ECM antigens could have influenced our results. The use of elastin peptides and lung collagen was based on previous data of Lee and Goswami *et al *[3,18] and the use of decorin on our previous results of diminished peribronchial decorin expression in COPD [17]. It can be envisaged that the neo-antigens that arise during the chronic inflammatory response in COPD differ in their protein conformation from ECM proteins expressed in normal lung tissue and that only neo-antigens can be recognized by the immune system. Because we induced antibodies against the original conformation of collagen and decorin and against elastin peptides, it is uncertain whether their cognate antigen epitopes are present in the lungs of smoking mice and whether these antibodies actually can evoke a local immune response.

## Conclusions

We showed that both ECM immunization and smoke exposure induced a specific immune response against ECM proteins and that ECM immunization itself increased macrophage numbers. The presence of a specific immune response against ECM proteins did not augment the smoke-induced inflammatory response in our model. This does not necessarily imply that there is no causal role for autoantibodies in augmenting the smoke-induced inflammatory response in COPD. The lack of an additive effect of autoantibodies and smoking in our study can well be due to a combination of a weak inflammatory response after smoking, the choice of antigens, and the fact that smoke exposure itself already caused an antibody response in our model. The development of additional autoimmune mouse models for COPD will be necessary to further investigate whether the specific immune response in COPD is a true autoimmune response contributing to the development of more severe disease.

## Competing interests

The authors declare that they have no competing interests.

## Authors' contributions

CB participated in the study design, performed the animal experiments, analyzed the data, performed statistical analysis and drafted the manuscript. MG and HJ carried out all the experimental analyses and were involved in the data analyses. WT, DS, MH and HK were involved in the study design and helped to draft the manuscript. All authors approved the final manuscript.

## Pre-publication history

The pre-publication history for this paper can be accessed here:

http://www.biomedcentral.com/1471-2466/10/64/prepub
